# “It’s how we get to know each other”: Substance use, connectedness, and sexual activity among men who have sex with men who are living with HIV

**DOI:** 10.1186/s12889-022-12778-w

**Published:** 2022-03-03

**Authors:** Amelia M. Stanton, Megan R. Wirtz, Jacob E. Perlson, Abigail W. Batchelder

**Affiliations:** 1 Department of Psychiatry, Massachusetts General Hospital, Behavioral Medicine, One Bowdoin Square, 7th Floor, Boston, MA 02114 USA; 2 Department of Psychiatry, Harvard Medical School, Boston, MA USA; 3 The Fenway Health Institute, Fenway Health, Boston, MA USA; 4 Geisel School of Medicine, Dartmouth College, Hanover, NH USA

**Keywords:** HIV, Men who have sex with men (MSM), Substance use, Sexuality, HIV

## Abstract

**Background:**

Among MSM, substance use increases risk for acquiring HIV and is associated with sub-optimal engagement in HIV-related care. Most research related to substance use and sexual activity among MSM focuses on identifying and reducing risk of HIV acquisition and transmission rather than pleasure and agency. However, substance use may also facilitate sexual pleasure and build community, which could be particularly meaningful for individuals who cope with intersecting stigmas related to the disease, sexual identity, and drug use.

**Methods:**

To explore the ways in which substance use both promotes and hinders positive sexual expression and healthy sexual relationships, we conducted a secondary analysis of 33 semi-structured qualitative interviews with MSM living with HIV who were poorly engaged in care and reported recent substance use.

**Results:**

Thematic analysis revealed that substance use was perceived as: (1) a potential pathway to intimacy and enhanced sexual experiences; (2) a tool to help access partners and gain entry to a community; and (3) a source of empowerment, though some noted that it sometimes came at the cost of sexual disempowerment and unbalanced relationships.

**Conclusions:**

Clinically, our results suggest that the complex motivations for substance use during sexual activity need to be carefully considered and discussed with patients, especially when attempting to decrease problematic use as a pathway to improved HIV self-care.

**Supplementary Information:**

The online version contains supplementary material available at 10.1186/s12889-022-12778-w.

## Introduction

Gay, bisexual, other men who have sex with men (MSM) are disproportionately affected by HIV infection in the United States (US), accounting for 70% of new HIV diagnoses each year (66% of which are attributed to exclusively male-to-male sexual contact, with an additional 4% attributed to both male-to-male sexual contact and injection drug use) [1, 2]. Engagement in HIV care, as well as early initiation of adherence to antiretroviral therapy (ART) to suppress viral load, improves health outcomes and effectively eliminates the risk of HIV transmission [3, 4]. However, only 57% of MSM living with HIV consistently attend HIV appointments, and, relatedly, only 58% are virally suppressed [5]. If MSM living with HIV are not actively attending appointments and regularly taking ART medications, they are not reaping the benefits of recent advancements in HIV treatment, including dramatically reduced HIV-related morbidity and mortality, and the assurance that undetectable = untransmittable (i.e., that individuals with an undetectable viral load do not sexually transmit HIV to others) [6, 7].

Active substance use has been identified as one of several barriers to HIV care engagement among MSM in the US. Substance use may reduce consistency of care, leading to missed appointments and other disruptions in the HIV care continuum [8]. Relatedly, substance use disorders are associated with decreased access to and use of health care services, reduced likelihood of being prescribed ART, and reduced adherence to ART [9]. Among MSM, stimulant use in particular has been associated with accelerated HIV progression and difficulties navigating HIV treatment, including maintaining consistent ART adherence [10–12]. Identifying motivators for use—both positive and negative—and developing a nuanced understanding of the benefits of substances may inform interventions to reduce problematic use and ultimately improve care engagement.

Motivations for substance use are complex and may differ by population, with minority stressors likely contributing to use among MSM. Several models have been developed to conceptualize motivations for use. Substance use has been characterized as a form of self-medication, such that use is initiated to decrease painful emotions, relieve psychological suffering, and maintain emotional stability [13]. Studies have both documented the comorbidity of mood disorders and methamphetamine (meth) use in MSM [14, 15] and suggested that childhood trauma, which is prevalent among MSM [16], may be associated with higher meth use in this population [17]. Meth and substance use generally may therefore function as self-medication to cope with low mood, other forms of emotional distress, and/or extensive trauma histories. The concept of minority stress [18] may also help explain substance use among MSM. Minority stress theory suggests that common identity-related negative experiences, including stigmatization and victimization, lead to a perceived need to conceal one’s sexual identity, which increases internalized biases that negatively impact mental health and lead to substance use as a form of coping [19, 20]. Both the self-medication hypothesis and the minority stress model suggest that substance use among MSM is driven by efforts to cope with negative emotions and experiences.

Substance use in the context of sexual activity, particularly among those living with HIV, may be catalyzed by other factors that warrant exploration. The literature on the positive motivations for and benefits of substance use during sexual activity in MSM remains limited and is even more sparse in MSM living with HIV, among whom substance use is discussed almost exclusively in relation to sexual risk behavior and HIV transmission. The few positive motivators for use that have been documented among MSM include increasing (1) desire for sexual activity, (2) intensity of a sexual experience, and (3) duration of a sexual experience [21]. So-called “party-and-play” or “chemsex” drugs, which may be used in the context of dance parties and nightclubs (e.g., crystal methamphetamine, ecstasy, ketamine, and gamma hydroxybutyrate/gamma-butyrolactone (GHB/GBL)), are known to increase libido and sexual stamina [22]. However, these are likely not the only positive motivators for use in the context of sexual activity, and the perceived benefits of use among MSM living with HIV have not yet been thoroughly assessed. It is possible, for example, that individuals living with multiple intersecting stigmatized identities (i.e., having HIV, using substances, being a man who has sex with men) may also use substances during sexual activity to build intimacy or strengthen connections that they struggle to find elsewhere [23, 24]. According to the intersectionality framework, multiple stigmatized identities (e.g., race, gender, socioeconomic status, sexual orientation, ability) interlock at the individual level and interact with systems of structural privilege and societal oppression [25–29]. Some suggest that an intersectional approach to stigma indicates that different forms of stigma can be synergistic and mutually constitutive, such that the effects of one minority stressor (e.g., HIV status) are dependent on those of another minority stressor (e.g., substance use), or vice versa [30–32]. Based on intersecting identities and/or co-occurring conditions, intersectional stigma may derive from multiple sources at multiple levels of influence [33]. Within this framework, those living with multiple stigmatized identities are likely at greatest risk of social isolation and/or rejection [34] as well as the associated negative health consequences [35, 36].

Although decades of research has documented elevated rates of drug and alcohol use, as well as the negative implications of use as it pertains to sexual activity and what has broadly been characterized as sexual risk behavior, positive relational motivators for use have not been widely explored among MSM with HIV, especially from an intersectional stigma framework. In this sub-analysis of a larger project that examined the ways in which intersecting stigmatized identities impact engagement in HIV care among MSM who use substances [37], we explored motivations for and potential benefits of substance use in the context of sexual activity, an emergent theme of the parent study. If providers are not aware of the benefits of substance use and are therefore unable to elicit and meaningfully address these sexual and relational motivations, efforts to reduce use, promote safer use, and improve HIV self-care may not resonate with patients.

## Methods

### Procedures for the parent study

MSM living with HIV who use substances were recruited through online websites and apps (e.g., Craigslist, Scruff, Facebook) as well as advertising/outreach at community settings and providers of substance use treatment, HIV care, and/or other support services [38]. Individuals who met the following inclusion criteria and provided written informed consent were eligible to participate in semi-structured qualitative interviews: (1) living with HIV; (2) recent substance use, including alcohol, assessed via questions from the Alcohol, Smoking, and Substance Use Involvement Screening Test (ASSIST [39]), though tobacco use alone was insufficient for inclusion; (3) being gay, bisexual, or otherwise a male who has male sexual partners; and (4) sub-optimal engagement in HIV self-care, defined as either having a detectable HIV RNA viral load, reporting < 90% antiretroviral adherence in the past month, or missing ≥ 2 HIV-related medical appointments in the past year without rescheduling them. Problematic substance use was assessed with questions from the Addiction Severity Index (ASI-LITE) but was not required for participation [40].

The protocol and interview guide [41]; please see attached supplementary file for the interview guide] from the parent study were approved by the Research Committee of the Fenway Institute (1,042,713). Interviews ranged from 30–75 min in length and were conducted by a trained interviewer in a private room. The semi-structured interview guide included questions that probed participants’ identity/ies in general, specific identities, substance use, HIV self-care behaviors, and relationships between these topics. Participants were first asked broad, open-ended questions about their identities; then, they were asked to describe the ways in which these identities and their experiences of intersecting stigmas affected their substance use, HIV self-care behaviors, and their self-perceptions. Motivations for substance use during sexual activity were not specifically assessed in the interviews, but themes related to this topic emerged in the data. Interviews were conducted until thematic saturation was reached.

### Analysis

Thematic analysis, [42] informed by grounded theory [43] and by the intersectionality framework, was used to identify themes and subthemes that emerged from the qualitative interview data in the parent study. All transcripts were open-coded; after an initial meeting to discuss the codes, the codes were collated into themes and sub-themes. Over several additional meetings to re-review the transcripts, the themes were iteratively refined and defined. A few transcripts were then double coded to again refine the thematic coding structure, and any discrepant codes were discussed and resolved. Finally, all 33 transcripts were double-coded by members of the team using the final coding structure. All primary analyses were conducted using NVivo version 12 [44].

To conduct the current analysis, we selected two themes from the original coding structure: (1) “substance use”, which was defined as “any content related to substance use”, and (2) “sexual orientation or behavior”, which was defined as “any content related to sexual orientation, sexual behavior, including bisexuality, and straight-identified MSM”. We reviewed all text that fell within these two codes, using content analysis to identify and refine subthemes related to specific motivations for substance use in the context of sexual activity. One team member (AMS) conceptualized three emergent motivations for substance use in relation to sexual activity and presented multiple examples of each motivation to a second team member (AWB), who conducted the interviews for the parent study. Among the two team members, discrepant interpretations were discussed and resolved by revisiting the original context for clarity. Quoted text that illustrates each of the three motivations are presented here.

## Results

### Sociodemographic characteristics

The final sample included 33 participants who all endorsed having sex with men and had a median age of 54 years (IQR = 12; range 26–68). Most participants identified as Black/African American (60.6%). With respect to educational level and socioeconomic status, 48.5% had graduated college or had some college, 36% of participants had a high school education or less, and the majority (75.7%) had an annual income of ≤ $20,000. Almost all participants reported recent alcohol use (90.9%), 78.8% indicated recent marijuana use, 60.6% endorsed recent cocaine/crack use, and 39.4% reported recent use of club drugs (defined as “drugs like ecstasy, ketamine, and GHB*”* in the assessment*)*. Rates of problematic use across all substances were lower than overall rates of use; 54.5% of participants reported problematic stimulant use (i.e., problematic use of either cocaine/crack or amphetamines), 36.4% reported problematic alcohol use (with 33.3% reporting problematic use of alcohol to intoxication), 12.1% reported problematic opioid use, 21.2% reported problematic marijuana use, and 45.5% reported problematic use of more than one substance. Please see Table 1 for demographic information and Table 2 for data on substances used by participants in the past three months [45]. Table 3 presents the number and associated percentages of participants who engaged in polysubstance use (i.e., presents the number of participants who used different combinations of any two drugs at the same time) in the past three months.

**Table 1:** Demographics (*n* = 33)

*Characteristics*		
Age (years)		
Median (interquartile range)	54	12
Range	26–68	
Years Living with HIV Median (interquartile range)	18	17
Range	0–38	
	n	%
Race		
Black/African American	20	60.6%
White	12	36.4%
Other	1	3%
Ethnicity		
Hispanic/Latino	1	3%
Education		
≤ Highschool graduate	12	36.4%
Some college/college graduate	16	48.5%
> College	5	15.2%
Sexual Orientation		
Gay/homosexual	19	57.6%
Bisexual	9	27.3%
Other^a^	5	15.2%
Income		
$10,000 or less	14	42.2%
$10,001 to $20,000	11	33.3%
$20,001 and above	8	24.2%

^a^Other includes both participants who selected “straight” and “other.”

**Table 2:** Substance use

*Substance Use in the Past 3 Months*^*a*^	*n*	%
Alcohol	30	90.9%
Club drugs (e.g., ecstasy, ketamine, GHB, or similar)	13	39.4%
Cocaine/crack	20	60.6%
Amphetamines	17	51.5%
Opioids	7	21.1%
Sedatives/ benzodiazepines	12	36.4%
Hallucinogens	1	3.0%
Marijuana	26	78.8%
Tobacco	25	75.8%

^a^Assessed using a modified version of the Alcohol, Smoking and Substance Involvement Screening Test (ASSIST; Babor, 2002)

**Table 3:** Polysubstance use

Number (percentage) of participants who used two substances at the same time in the past three months, by substance									
	*Alcohol*	*Club Drugs*	*Cocaine/Crack*	*Amphetamines*	*Opioids*	*Sedatives/* *Benzodiazepines*	*Hallucinogens*	*Marijuana*	*Tobacco*
*Alcohol*	–	10 (30.3)	18 (54.5)	14 (42.4)	7 (21.2)	10 (30.3)	1 (3.0)	24 (72.7)	23 (69.7)
*Club Drugs*	10 (30.3)	–	7 (21.2)	13 (39.4)	2 (6.1)	6 (18.2)	0 (0.0)	11 (33.3)	11 (33.3)
*Cocaine/Crack*	18 (54.5)	7 (21.2)	–	11 (33.3)	5 (15.2)	8 (24.2)	0 (0.0)	16 (48.5)	16 (48.5)
*Amphetamines*	14 (42.4)	13 (39.4)	11 (33.3)	–	3 (9.1)	8 (24.2)	0 (0.0)	15 (45.5)	14 (42.4)
*Opioids*	7 (21.2)	2 (6.1)	5 (15.2)	3 (9.1)	–	5 (15.2)	1 (3.0)	6 (18.2)	7 (21.2)
*Sedatives/Benzodiazepines*	10 (30.3)	6 (18.2)	8 (24.2)	8 (24.2)	5 (15.2)	–	1 (3.0)	9 (27.3)	11 (33.3)
*Hallucinogens*	1 (3.0)	0 (0.0)	0 (0.0)	0 (0.0)	1 (3.0)	1 (3.0)	–	1 (3.0)	1 (3.0)
*Marijuana*	24 (72.7)	11 (33.3)	16 (48.5)	15 (45.5)	6 (18.2)	9 (27.3)	1 (3.0)	–	16 (48.5)
*Tobacco*	23 (69.7)	11 (33.3)	16 (48.5)	14 (42.4)	7 (21.2)	11 (33.3)	1 (3.0)	16 (48.5)	–

### Overview of results

The majority of participants reported content that was coded as either “substance use” or “sexual behavior” or content that was coded under both categories. About half of participants described complex motivations for substance use and some notable negative consequences of using substances in the context of sexual activity. Three key motivators for substance use during sexual activity emerged from the data. Participants conveyed that substance use (1) offers a potential pathway to intimacy and enhanced sexual experiences; (2) grants access to partners and community, in ways that can be both affirming and harmful; and (3) leads to a sense of empowerment that enables vulnerability and allows for sexual choices that might otherwise lead to discomfort and shame. Others described how substance use was disempowering in certain contexts and created power imbalances that may not be conducive to healthy relationships. Although substance use in anticipation of or during sexual activity was often fueled by the desire to access partners and have positive sexual experiences, participants reported that substance use also contributed to fleeting and ungratifying friendships, verbal abuse, and sexual relationships that stripped them of their agency and power, ultimately leading to rejection in some cases.

### A pathway to intimacy and enhanced sexual experiences

Several participants explained the ways in which substance use builds intimacy; enhances sexual experiences, such that use decreased inhibitions; and enabled positive or “optimal” interactions. By improving dyadic aspects of the sexual experience, participants described the ways in which substance use supported relationship growth and development. One participant reported that substance use enabled him and his partner not only to become more intimate in a sexual context, but also to learn about each other, strengthening their relationship:

*Yeah, [we] do them together; we drink, smoke, take another drink…and cigarettes. So all of that is there… It’s the way that he and I are intimate with each other, it’s just the two of us, it’s how we get to know each other, get to enjoy each other.* (Black Male)

Another participant expressed a similar sentiment, noting that substance use was often motivated by the possibility of easing interpersonal interactions and ultimately improving sexual experiences. He describes a thoughtful effort to use just enough to optimize a given sexual encounter:

*I do the drug to, in essence, to enhance the sexual situation...Well, kind of, you know, like, social drinking. Like, getting to that point where you have that pretty cool buzz, and you don’t go over that.* (White male)’

Although substance use was described as a means to more intimate or enhanced sexual experiences, participants reported that using in the context of sexual activity sometimes separated them from their own reality, creating a conditional or transient sense of happiness. To some, this sense of detachment created tension when they readjusted to social functioning in the absence of substances. One participant reported that the separation made the return to “real people” in “real situations” that much more difficult:

*When I’m with people who are using and having sex I’m having the best time. But if I have to go out in public, and either high or under the influence, yeah, I don’t feel so good about myself. Because then I have to face real people in real situations.* (White male)

Although substance use during sexual activity enabled enhanced sexual experiences and therefore a welcomed departure from reality, participants indicated that it also robbed them of the positive experiences that are neither associated with substance use nor sexual behavior. One participant verbalized this concern, noting that time passed while he was using and that he was not able to participate in activities that he enjoys:

*When you’re doing this stuff, you’re basically focused on sex and having fun and porn. And you’re in dark rooms or hotel rooms or some back bedroom. And there’s so much of life you miss. There’s so many things I don’t do...I love going to the beach and Six Flags and rollercoasters. I love rollercoasters…We’ve had whole summers go by and we don’t do anything, because we were too busy partying.* (White male)

Overall, participants reported using substances in the context of sexual activity to facilitate intimacy and enhance sexual experiences. For some, these heightened sexual interactions came at the cost of difficulty attending to the present moment. This was’ both appreciated as a momentary reprieve and also recognized as a negative byproduct of use.

### Access to partners and community

Participants described substance use as a tool for accessing partners and building different types of relationships, both with individual sexual partners and with members of their community in “party-and-play” or “chemsex” contexts. Yet, for some, the use of substances to access partners was often associated with some manifestation of sexual or relationship imbalance. One participant spoke to the positive impacts of substance use in broadening his social network and bolstering a sense of desirability. He reported a relationship among substance use, sexual activity, and engaging with highly attractive sexual partners that he perceived to be inaccessible.

*I was hanging with these guys at their house, visiting, and everybody kept disappearing…They were drug dealers, but I didn’t know that…“Oh, have you ever tried it?” “No, how do you do it?” They go, “You can have anything you want – have anybody, do anything you want.” So, it’s all these gorgeous, masculine guys coming in later on, and they want to get high...“You can have them do anything you want.”…So that’s how I learned about having drugs and sex.* (Black male)

For another participant, substance use was necessary to increase the frequency of sexual activity within a monogamous relationship. This participant also spoke to the potential for substances to create substance-related imbalances in sexual relationships, such that one partner wanted to engage or was already using in the context of sex, whereas the other partner may not have been using at that time. For this participant, being out of sync with his partner with regards to substance use lead to a tense and sexually unfulfilling relationship.

*So, I have dated someone; we met at a sex party, but when we started dating, he was clean, and maybe in 9 months we had s’ex three times, and it just drove me nuts. So, for him, he needed it.* (White male)

Substance use in the context of sexual activity was also discussed from the perspective of the community, revealing that some participants perceived substance use to be an entry point and/or an opportunity to gain understanding and acceptance from others. One participant conveyed the sentiment that, before he tried crystal meth, he felt like an observer or “monitor” of gay culture rather than an active and informed community member.

*I just felt like I, when I was doing the gay marriage thing, and canvassing and things…I didn’t want to be, like, the hall monitor…Because I didn’t know enough. I didn’t want it to pass me by because I wouldn’t have felt like I was part of the community.* (White male)

He continued to explain that he felt the need to share in the experience of crystal methamphetamine to understand and participate in the community. This feeling was particularly salient in the context of meeting new people or affiliating with a new group; if members of a new group were using substances and engaging with each other around those substances, the participant wanted to be able to share in that experience. He did not want to act as a “hall monitor” or a superficial participant that was not really part of the group, in which others were being “taken down” by meth. Therefore, he reported grappling with the decision to become involved in the methamphetamine scene:

“And if…I’ve never even met anyone in that circle and I don’t know what they’re talkingabout, and they’re, like, taking down – I’m not being taken down. Like, who is getting taken down by who? Crystal meth? Who’s that? That’s why I did it. (White male)

The same participant described his frustrations with his substance use “path” but noted that, in addition to using for the purpose of understanding and taking part in the community, he also used to secure a committed, long-term partner.

*My path that I’ve been on, I’m not kidding…was to find a husband.* (White male)

In sum, participants indicated that they used substances during sexual activity to gain access to certain partners, to maintain relationships with partners, and to increase knowledge of and participation in their community. Some described the association among substance use, sex, and partnership or community with frustration or resignation, suggesting challenges initiating and maintaining relationships in which substances play a significant role.

### Empowerment/disempowerment

Substance use during sexual activity was described by many as motivated by the desire for self-expression or relief from the pressures of identity concealment. Participants reported that decreased pressure to conceal their identities empowered them to engage in specific sexual acts with certain partners that were previously linked with feelings of vulnerability. In text that was quoted earlier, a participant noted that substance use enabled access to “gorgeous masculine guys”, men that may otherwise have been too intimidating to approach. This statement exemplifies sentiments shared across participants. Indeed, another participant reported that substance use prior to or during sexual activity enabled same-sex sexual experiences that might seem shameful under different circumstances. He conveyed the notion that substance use in the context of sexual activity offered the opportunity for others to be “themselves”, that is, to be more “truthful” in expressing their sexuality.

*I have to laugh as soon as they say they’re not gay, but they want to come to your house to get high. As soon as they take a hit, they’re the ones whose clothes come off automatically…Like, some guys can smoke weed or coke…, but they’re using that as… a cover-up. “Oh, I was high,” or “I was drunk.” OK, so that’s just the truth drug. You’re just being yourself then.* (Black male)

Though substance use during sex was described as empowering some to be vulnerable, enact their sexual desires, and explore stigmatized identities, multiple participants indicated that substances led to different forms of disempowerment, particularly within a dyad or group. For instance, some described substances as a tool to appease sexual partners, enabling the partners to have the enhanced sexual experiences that they were seeking, but often leaving the participants feeling used. One participant indicated that he used substances to ensure that others had these optimal experiences, intimating that he may not have reaped the same benefits or enjoyed the experience to the same degree:

*And, it’s progressed to the point that the person also -- and these people, in many instances, they use drugs -- crack cocaine -- they drink, they smoke weed, and they want you to indulge in these behaviors to make the experience optimal what they want. And so, I’ve done that.* (Black male)

Another participant described a similar sentiment, highlighting that he engaged with substances in the context of sexual activity to avoid being alone. Substance use in this context may also act as a form of self-medication to cope with the negative emotions (e.g., shame, sadness, anger) and cognitions that are associated with isolation, which may itself be a strategy to avoid distal and proximal sexual minority stressors. Ultimately, the participant found little pleasure for himself and was left feeling both shame and used by others:

*Because sometimes, you know, that whole feeling numb thing is so much better than being alone, so you kind of put yourself in positions where you’re being taken advantage of for the sex and you’re not even enjoying it. But I think that’s why in the last month-and-a-half that I have been really disgusted with a lot of my ways.* (White male)

Indeed, several other participants reported that substance use during sexual activity led to brief or conditional friendships and relationships that ended in rejection and abuse. For example:

*You could have sex with someone, and use for one night, and – or for five – you could use for a year. And then, the next day, you call them, and they say, “We don’t want you. F you. We don’t want you anymore.” Click.* (White male)

Again, though participants reported that substance use was motivated by the promise of intimacy, enhanced sexual interactions, access to partners/community, and increased power to express one’s sexual desires with decreased shame, they also noted that substance use sometimes led to unstable, transient relationships. Participants described examples of the ways in which substance use in the context of sexual activity disempowered them, leading to self-disgust and loneliness.

## Discussion

In this analysis, we explored emergent themes related to the ways in which substance use during sexual activity promotes and hinders positive sexual expression and healthy sexual relationships among MSM living with HIV. Although studies have addressed other interrelated motives for substance use (e.g., self-medication, coping with minority stressors [46, 47]), with a small subset of published work describing the ways in which substance use during sexual activity can facilitate social connectedness and a sense of belonging among MSM [48–50], few have (1) examined positive sexual or relational motivations for substance use in MSM living with HIV from a qualitative perspective, (2) described substance use in the context of intersectional stigma, and (3) discussed the implications of these motivations for clinical care [46, 48, 49, 51].

In a population with multiple intersecting stigmatized identities (i.e., having HIV, using substances, being a man who has sex with men), the importance of positive sexual expression and brief departures from the challenges of everyday life (e.g., stigma, social isolation) cannot be understated, even as these motivations do not negate the potentially destructive effects of harmful or problematic use. In aggregate, the three main themes that emerged from the data (pathway to intimacy and enhanced sexual experiences, access to partners/community, and empowerment/disempowerment) share a focus on interpersonal interactions and relationships, indicating that substance use in the context of sexual activity may be motivated by relational needs, among them identity affirmation and belongingness. The potential loss of intimacy, pleasure, partners, community, or power within a dyad or group may reinforce the internalized stigma or shame that is commonly reported in this population, potentially reducing engagement in HIV care [51, 52].

Most of the published literature on motivations for substance use during sex among MSM has focused on so-called “chemsex” drugs (e.g., amphetamines, cocaine, and club drugs, such as ecstasy, ketamine, GHB, or similar) as facilitators of sex with multiple partners either in sequence or as a group, not on the use of other drugs, like alcohol and marijuana, which may be associated with different motivating factors, particularly among individuals with multiple intersecting stigmatized identities. In addition to the described use of stimulants (amphetamines and cocaine) and club drugs (such as ecstasy, ketamine, and GHB), participants in this study discussed drinking alcohol and smoking marijuana to build intimacy, decrease initial discomfort (e.g., as in “social drinking”), or enhance sexual experiences with single or primary partners, not necessarily with multiple partners or in a group sex context. Certain drugs may have specific positive effects that MSM actively seek [22, 53]; some have suggested that alcohol and cannabis may increase motivation to have sex, whereas others, like stimulants and club drugs, are used to enhance pleasure [21]. Our findings suggest that a broader range of substances may be used to addressed the unique barriers experience by MSM with HIV, including initiating sexual activity, especially when in the early stages of a relationship with a potential partner. Exploring the specific ways in which intersecting stigmatized identities (e.g., having HIV, using substances, being a man who has sex with men) may influence alcohol, marijuana, and other substance use (i.e., beyond stimulants and club drugs) is an important area for future research.

Our findings do also address positive motivations for using substances that are more often discussed in the literature, providing some context for recently published quantitative data on the association between substance use, including crystal methamphetamine, and wellbeing among gay and bisexual men living with HIV in Australia [48]. Compared with persons who do not use crystal methamphetamine and other chemsex drugs substances, individuals who do use these substances reported higher levels of resilience and lower levels of perceived HIV-related stigma [48]. The authors suggested that, though these drugs may pose health risks, the social contexts in which they are used may improve well-being, particularly for those who are subjected to HIV- or sexual orientation-related stigma in other settings. In another secondary analysis published on the same qualitative data as the present study, the authors concluded that stigma related to substance use was a major contributor to the social isolation and self-critical internal states that are associated with managing multiple concealable stigmatized identities [45, 54]. In other studies of MSM who did not have HIV, substances, and the use of party-and-play or chemsex drugs including crystal methamphetamine and other club drugs such as ecstacy, ketamine, and GHB, in particular, acted as a point of entry into gay friendly and affirming social spaces where MSM could interact with peers and express their sexuality without fear of stigma and social disapproval [22, 55]. Within the framework of sex-based sociality [23], relationships, drug use, and sexual practice are mutually reinforcing and integral to the self-identity of sexual minority men as a marginalized group [23, 56]. Therefore, sexual intimacy, social support, and social connectedness may lessen the negative effects of any additional stigma among MSM living with HIV.

Regarding substance use as globally negative obscures the ways in which use can, despite the risks, result in sexual wellbeing benefits, especially for MSM living with HIV. Providers caring for this population may consider operating from a sexual wellbeing perspective. Although the term “sexual wellbeing” has been challenging to define [57], the term generally refers to an individual’s subjective assessment of a wide range of physical, cognitive, emotional, and social aspects of sexual relationships [58]. More specifically, others have suggested that sexual wellbeing encompasses concepts like sexual self-esteem; sexual agency; feelings of arousal, satisfaction, and pleasure; and freedom from pain, anxiety, and negative affect regarding sexuality [59]. Rather than encouraging MSM living with HIV to reduce their substance use (or stop using altogether), providers should instead acknowledge and discuss the complex and multifaceted benefits of use with their patients. After doing so, they can work with patients to engage in harm reduction strategies to reduce or replace substance use with other pathways to the sexual wellbeing and intimacy benefits described by participants in this study, pathways that may decrease the intensity of the negative consequences that were also described by participants (e.g., disempowerment within partnerships). Such an approach reflects an affirming framework that may be characteristic of maximally effective and culturally relevant sexual healthcare [60].

This approach also aligns with the principles of harm reduction, which refers to reducing the negative effects of certain health behaviors without necessarily stopping the behavior completely or permanently. Using the harm reduction framework, providers identify the negative consequences of substance use as the target for intervention rather than substance use itself, recognizing that use in and of itself does not equal harm [61, 62]. Importantly, abstinence is neither prioritized nor assumed to be the goal of the patient [62]. As there is no single formula or strategy for implementing harm reduction across clinics or within specific groups, harm-reduction informed approaches can focus on unique community needs [63]. For MSM living with HIV, fostering intimate relationships and building connections may help mitigate the negative effects of sexual minority stressors and intersecting stigmatized identities. That is, among MSM living with HIV, a harm reduction approach will acknowledge the ways in which substance use during sexual activity may increase self-esteem and sexual agency, intensify feelings of arousal and satisfaction, and provide temporary relief from pain, while also working toward incremental changes that will favor benefits over harms.

There are several limitations of this study that are worth noting. Though the parent study reached thematic saturation with 33 interviews, the sample of MSM living with HIV was heterogenous with respect to type of substance use, substance use severity, and sexual orientation (i.e., men included in the sample identified as gay, bisexual, and straight). Therefore, given these variabilities, it is possible that motivations for substance use in the context of sexual activity differ by substance use type, degree of substance use severity, and sexual orientation. Importantly, the sample had a fairly diverse age range, but the majority of the men included in the study were over age 50. The results may have differed if the sample had included more younger men. Furthermore, the parent study did not assess the timing of substance use initiation relative to participants’ HIV diagnoses. Substance use may have preceded the HIV diagnosis or vice versa, potentially influencing motivations for use during sexual activity. Finally, there are inherent limitations associated with conducting a secondary data analysis that should be acknowledged. In the interview guide developed for the parent study, specific questions probing positive motivations for substance use were not included. Therefore, the motivations described here are likely not comprehensive; had all participants been asked to describe their reasons for using substances in the context of sexual activity, we would have been able to assess which motivations were most salient. Relatedly, because participants were not asked to indicate which drugs they used in preparation for and during sexual activity, we do not have quantitative data on the specific substances that were used in a sexualized context. These data would have provided adjunctive information on the nature of sexualized drug use across substances, highlighting that substance use during sex is not exclusive to stimulants, which is evident in specific references to alcohol, marijuana, and other drug use within the sample. Though the inductive nature of these analyses is somewhat limiting, themes related to motivations for substance use during sexual activity came up organically during the interviews, without probing, which indicates that this topic is likely salient and clinically meaningful to this population.

## Conclusions

In sum, this sub-analysis offers insights on positive and complex motivations for substance use in the context of sexual activity among MSM living with HIV. Given these findings, providers may recognize the relational challenges faced by this population. To support the sexual wellbeing of MSM living with HIV, they may choose to assist patients who want to reduce their substance use (and/or stop using) in finding alternative sources of sexual pleasure, community, and empowerment that do not compromise connectedness, intimacy, or agency. Attending to the sexual wellness of MSM living with HIV does not trivialize the HIV transmission risk associated with condomless sex, though it does encourage an acknowledgement of the stigma-reducing potential of the “undetectable = untransmittable” campaign [64]. Acknowledging the importance of sexual wellbeing means (1) proactively asking patients about their sexual activity, (2) inquiring about potential sexual concerns, (3) demonstrating willingness to help patients navigate those concerns, and (4) most importantly, accepting (and verbalizing) that healthy sexuality among MSM and people living with HIV involves more than just reducing transmission and avoiding other commonly invoked negative consequences.

## Supplementary Information

**Additional file 1.** Intersecting Stigmatized Identities & Engagement in HIV Treatment (INSIGHT) Study.

## Data Availability

The datasets generated and/or analyzed during the current study are not publicly available due the possibility that sharing these interviews, which contain sensitive information about participants’ identities, may compromise participant anonymity; however, the data are available from the corresponding author on reasonable request.

## Abbreviations

ART: Antiretroviral therapy
ASI-LITE: Addiction Severity Index
ASSIST: Alcohol, Smoking, and Substance Use Involvement Screening Test
GHB/GBL: Gamma hydroxybutyrate/gamma-butyrolactone
HIV: Human immunodeficiency virus
MSM: Men who have sex with men
RNA: Ribonucleic acid
