# Supplementary material for: “It’s how we get to know each other”: Substance use, connectedness, and sexual activity among men who have sex with men who are living with HIV
Source: BMC Public Health. 2022 Mar 3;22:425. doi: 10.1186/s12889-022-12778-w (PMC8895830; doi:10.1186/s12889-022-12778-w)
Supplement: Supplementary file 1 — Additional file 1. Intersecting Stigmatized Identities & Engagement in HIV Treatment (INSIGHT) Study. [file 12889_2022_12778_MOESM1_ESM.docx]

Intersecting Stigmatized Identities & Engagement in HIV Treatment (INSIGHT) Study

*I am interested in learning more about how you think and feel about yourself and if that impacts your self-care. I am very interested in hearing what you have to say, there are no right or wrong answers. This interview will take between 60-90 minutes and if you need a break, just let me know and I will stop. Everything you tell me today will be kept confidential, and will not affect your treatment here or at other clinic sites. I will tape-record this session, but your name will not be on the tape, just a visit number.  After the session, the tape will be typed up into a transcript, and the original tape will be erased.  If you happen to refer to a name during the session, the name will be disguised in the transcript. The only people who will see this transcript are involved in this project.*

*Any time you want to stop the interview or have me turn off the tape, you can tell me and we will stop.*

First, I’d like to ask you about your HIV-related self-care. How is that going?

Are you taking your ART medications daily?

If not, why do you think you’ve missed doses?

*[Probe as necessary]*

Are you making it to your HIV-related appointments?

If not, why do you think you’ve missed appointments?

*[Probe as necessary]*

What gets in the way of your HIV-related self-care?

*[Probe as necessary]*

How do you think about yourself?

How would you describe yourself to someone who didn’t know you (and couldn’t see you)?

*(probe: age, race, ethnicity, sexual orientation)*

Do you think about yourself as a person living with HIV?

Do you think about yourself as a person who uses substances?

Does the way you think about yourself ever impact your behavior(s)?

How about self-care behaviors?

*[Probe as necessary]*

Do you think/feel like substance use impacts your HIV-related self-care?

If so, how?

Do you think/feel like aspects of your identity (or the way you think about yourself) are related to the way you think about being HIV+?

If so, how? *[Probe as necessary]*

Being MSM?

Being a person who uses substances?

Do parts of your identity seem to be related to one another?

*[Probe as necessary]*

What else impacts your HIV-related self-care?

Do you ever feel like you are avoiding HIV-related self-care?

If yes, then how so?

Why do you think that is?

Is there anything else you’d like to share?

Do you have any additional thoughts about how stigma may impact your self-care?

Thank you for your time and for your thoughts. We really appreciate you sharing your perspective.

Notes:
